# Selective HDAC6 Inhibition Has the Potential for Anti-Cancer Effect in Renal Cell Carcinoma

**DOI:** 10.3390/jpm14070704

**Published:** 2024-06-30

**Authors:** Tsutomu Anraku, Masaki Murata, Hiroo Kuroki, Akira Kazama, Yuko Shirono, Masayuki Tasaki, Vladimir Bilim, Yoshihiko Tomita

**Affiliations:** 1 Department of Urology, Division of Molecular Oncology, Graduate School of Medical and Dental Sciences, Niigata University, Niigata 951-8510, Japan; m-murata@med.niigata-u.ac.jp (M.M.); hiroo0823@yahoo.co.jp (H.K.); exfeel@live.jp (A.K.); yuko-shirono@med.niigata-u.ac.jp (Y.S.); masa1214@med.niigata-u.ac.jp (M.T.); vbilim@zoho.com (V.B.); ytomita@med.niigata-u.ac.jp (Y.T.); 2 Department of Urology, Kameda Daiichi Hospital, Niigata 950-0165, Japan

**Keywords:** histone deacetylase 6, renal cell carcinoma, anticancer, apoptosis

## Abstract

Despite significant advancements in systemic therapy for renal cell carcinoma (RCC), the prognosis for patients with metastatic RCC remains poor, as they are often incurable. Consequently, there is an urgent need for innovative therapeutic strategies to further enhance the efficacy of RCC treatment and improve patient outcomes. One such promising avenue lies in targeting histone deacetylase (HDAC) 6, a protein known to regulate numerous crucial biological processes implicated in cancer progression by modulating the acetylation status of various cytoplasmic proteins. To explore the therapeutic potential of HDAC6 inhibition in RCC, our study focused on investigating the effects of HDAC6 inhibitors on cultured RCC cells. Utilizing a panel of 12 small molecule selective HDAC6 inhibitors and employing genetic knockdown techniques, we examined the impact of HDAC6 inhibition on RCC cellular dynamics. Our findings revealed that HDAC6 inhibition exerted a profound effect on RCC cells, resulting in decreased cell viability and DNA replication. Importantly, this effect was attributed to the induction of apoptosis. Our study provides valuable insights into the mechanisms underlying the anticancer effects of selective HDAC6 inhibitors on RCC. A detailed understanding of the molecular mechanisms underlying the anticancer effects of HDAC6 inhibition is important to explore new therapeutic strategies for metastatic RCC.

## 1. Introduction

Renal cell carcinoma (RCC) stands as the predominant form of renal malignancies, constituting a significant portion of cancer diagnoses globally. In the United States, RCC ranks as the 6th most commonly diagnosed cancer in men and the 7th in women, comprising 5% and 3% of all oncological cases, respectively [1]. Furthermore, it emerges as the 9th leading cause of cancer-related deaths, with more than 14,000 deaths each year in the United States.

Over the past two decades, the landscape of systemic therapy for metastatic RCC has undergone remarkable changes, transitioning from the traditional use of immunotherapeutic interferon-α to a variety of molecularly targeted drugs and immune checkpoint inhibitors. Despite these advancements, the prognosis for most metastatic RCC cases remains poor, with a median progression-free survival of 2 years or less for patients subjected to first-line combination therapy involving molecularly targeted drugs and immune checkpoint inhibitors [2,3,4,5,6]. Notably, the combination of lenvatinib and pembrolizumab has demonstrated the highest complete response rate among these therapeutic regimens, although it remains only approximately 16% [4]. Therefore, the quest for novel therapeutic targets within the realm of RCC has become an urgent imperative.

Histone acetylation and deacetylation are crucial components of the epigenetic mechanisms that control gene expression [7]. Elevated histone acetylation is linked to enhanced transcriptional activity, while reduced acetylation correlates with repression of gene expression [8]. Histone deacetylase (HDAC) is an enzyme that plays a crucial role in the epigenetic regulation of gene expression through histone deacetylation [9]. Within the HDAC family, comprising 18 members, HDAC6 emerges as a unique entity, predominantly localizing in the cytoplasm. Unlike its counterparts, HDAC6 targets cytoplasmic proteins for acetylation, thereby regulating numerous biological processes pivotal for cancer initiation, promotion, proliferation, metastasis, and the maintenance of cancer stem cells [10,11]. Overexpression of HDAC6 has been reported in various cancers, and some HDAC6 inhibitors are in clinical trials for the treatment of cancer [12,13,14,15,16,17]. The rationale behind targeting HDAC6 lies in its unique role in regulating cytoplasmic proteins, which are crucial for various aspects of cancer biology. Additionally, HDAC6 exhibits a distinctive structure, including two catalytic domains, making it suitable for the development of selective inhibitors [17]. Consequently, the inhibition of HDAC6 represents a promising avenue for novel anticancer strategies.

In this study, we elucidate the anticancer properties of selective HDAC6 inhibitors in renal cancer cell lines, shedding light on their potential therapeutic efficacy and paving the way for further exploration into their clinical application.

## 2. Materials and Methods

### 2.1. Immunohistochemistry

We purchased tissue microarray KD483 from US Biomax, which contained 27 RCC specimens. Immunohistochemical staining was performed as follows: After deparaffinization of paraffin tissue sections with xylene and ethanol, epitopes were reactivated by autoclaving in 10 mM citrate buffer (pH 6.0) for 20 min at 120 °C. The slides were then incubated overnight at 4 °C with the anti-HDAC6 antibody (Cell Signaling Technology, catalog number 7558). Subsequently, they were treated with Histofine Simple Stain MAX-PO (Nichirei, Tokyo, Japan) for 30 min at room temperature. Staining reactions were visualized using 3,3′-diaminobenzidine, followed by nuclear counterstaining with Mayer-Hematoxylin Solution (Fujifilm, Osaka, Japan). Staining intensity was graded as follows: 0, no staining; 1, weak staining; 2, moderate staining; and 3, strong staining. The proportion of stained cells was categorized as follows: 0, 0–4%; 1, 5–24%; 2, 25–50%; and 3, 50–100%. Immunohistochemical staining scores were determined by multiplying the intensity score by the proportion score. Low and high expression were defined by the scores between 0 and 4 and 6 and 12, respectively.

### 2.2. Cell Culture and Reagents

The RCC cell lines ACHN, A498, Caki-2, and KRCY were sourced from the American Type Culture Collection, while Caki-1 was acquired from the Japanese collection of Research Bioresources Cell Bank. KU19-20 was generously provided by Dr. Mototsugu Oya from the Department of Urology, School of Medicine, Keio University, Tokyo, Japan. The cells were maintained in RPMI-1640 medium (Gibco; Thermo Fisher Scientific, Inc., Grand Island, NY, USA) supplemented with 10% Fetal Bovine Serum (Gibco; Thermo Fisher Scientific, Inc.), 1% MEM Non-Essential Amino Acids (Gibco; Thermo Fisher Scientific, Inc.), 1% MEM sodium pyruvate solution 100 mM (Gibco; Thermo Fisher Scientific, Inc.), and 90 μg/mL kanamycin in a 37 °C incubator with 5% CO_2_. In the case of mycoplasma contamination, the cells were treated with MC-210 (DS Pharma Promo Co., Ltd., Osaka, Japan) at 0.5 μg/mL for 2 weeks and cultured in RPMI medium without MC-210 for another week before experiments. Selective HDAC6 inhibitors were provided by the Department of Medicinal Chemistry and Pharmacognosy, University of Illinois at Chicago.

### 2.3. Cell Viability and DNA Replication Assays

Cell viability and DNA replication in S phase were assessed using a colorimetric CellTiter 96^®^ AQueous One Solution Cell Proliferation assay (Promega Corporation, Madison, WI, USA) and a 5-bromo-2-deoxyuridine (BrdU) Cell Proliferation Assay kit (EMD Millipore, Bolington, MA, USA), respectively, following the manufacturer’s protocol. In the cell viability assay (MTS assay) and DNA replication assay (BrdU incorporation assay), 2 × 10^3^ cells/well (2 × 10^4^ cells/mL) and 5 × 10^4^ cells/well (5 × 10^5^ cells/mL) were seeded into flat-bottom 96-well plates (Corning, Corning, NY, USA), respectively. After confirming cell attachment, reagents were added. For the MTS assay, measurements were taken at 24, 48, 72, and 96 h. For the BrdU incorporation assay, BrdU was added after 48 h, and measurements were taken after an additional 24 h. Both experiments were performed in three or four replicates. Absorbance was measured at 490 nm in the MTS assay and at 450–595 nm in the BrdU incorporation assay using an iMark^TM^ 96-well microplate reader (Bio-Rad Laboratories, Inc., Hercules, CA, USA). The IC50, defined as the drug concentration that reduces cancer cell viability by 50%, was calculated using GraphPad Prism 7.

### 2.4. Analysis of the Cell Cycle and Apoptosis

Propidium iodide (PI) flow cytometry analyses were conducted using the FxCycle™ PI/RNase Staining Solution (Thermo Fisher Scientific) following the manufacturer’s protocol. Briefly, 1 × 10^6^ cells were harvested and fixed with cold 70% ethanol for 30 min. Subsequently, the cells were washed with PBS and incubated with PI/RNase Staining Solution at room temperature for 30 min, protected from light. For Annexin V/PI double staining, the MEBCYTO^®^ Apoptosis Kit was used following the manufacturer’s protocol. Briefly, 1 × 10^6^ cells were washed with PBS and resuspended in 85 μL of binding buffer. The cell suspension was then incubated with 10 μL of Annexin V-FITC and 5 μL of PI at room temperature for 15 min, protected from light. Following incubation, 400 μL of binding buffer was added. The stained cells were analyzed using the BD Accuri™ C6 Flow Cytometer (BD Biosciences, Franklin Lakes, NJ, USA) in both experiments.

### 2.5. siRNA Transfection

siRNA-mediated silencing was performed by forward transfection according to the protocol provided by Thermo Fisher Scientific. Briefly, cells were seeded and cultured in RPMI medium without antibiotics or fetal bovine serum. After 6 h, cells were transfected with 10 nM of siRNA targeting human HDAC6 (Thermo Fisher Scientific. siRNA ID 120452, Cat. No. AM51331) or 10 nM of negative control siRNA (Thermo Fisher Scientific. Silencer^®^ Negative Control #1 siRNA, Cat. No. AM4611) in RPMI medium without antibiotics or fetal bovine serum using Lipofectamine^TM^ RNAiMAX (Thermo Fisher Scientific, Cat. No. 13778-150). Cells were incubated for 24, 48, 72, and 96 h in antibiotic-free RPMI medium before further experiments.

### 2.6. Western Blot Analysis

Immunoblotting was performed as described previously [18]. Briefly, after lysis of cells using lysis buffer (150 mM sodium chloride, 100 mM Tris-HCl, 5 mM EDTA, 1% Triton X-100, and a protease inhibitor), the lysate was clarified by centrifugation at 15,000× *g* for 30 min. The protein concentration was measured using the Bradford method, and each protein sample (30 μg) was separated on a 10% SDS-polyacrylamide gel and transblotted to a PVDF membrane. The membrane was incubated overnight at 4 °C with the primary antibody, followed by a one-hour incubation at room temperature with a horseradish peroxidase-conjugated secondary antibody. Subsequently, immunoreactive bands were detected using Clarity Max Western ECL Substrate (Bio-Rad Laboratories) and analyzed with Ez-capture MG (Atto Corporation, Tokyo, Japan). The expression of β-actin or α-tubulin was used as a loading control. The primary antibodies used in the study were as follows: anti-HDAC6 (cat. no. 7558), anti-β-actin (cat. no. 12262), anti-α-tubulin (cat. no. 3873), and anti-acetyl-α-tubulin (cat. no. 5335) from Cell Signaling Technology. ECL^TM^ anti-mouse IgG (NA931) and ECL^TM^ anti-rabbit IgG (NA934) from GE Healthcare were used as secondary antibodies.

### 2.7. Statistical Analysis

Continuous variables are displayed as means ± standard deviation. All continuous variables conformed to a normal distribution and were treated as parametric. Data analysis was conducted using a one-way ANOVA with Dunnett’s test for multiple comparisons. Statistical analyses were performed using Prism 7 software (GraphPad Software, Inc., San Diego, CA, USA). *p*-values < 0.05 were deemed statistically significant.

## 3. Results

### 3.1. HDAC6 Was Expressed Predominantly in the Cytoplasm of RCC

**Figure 1:**
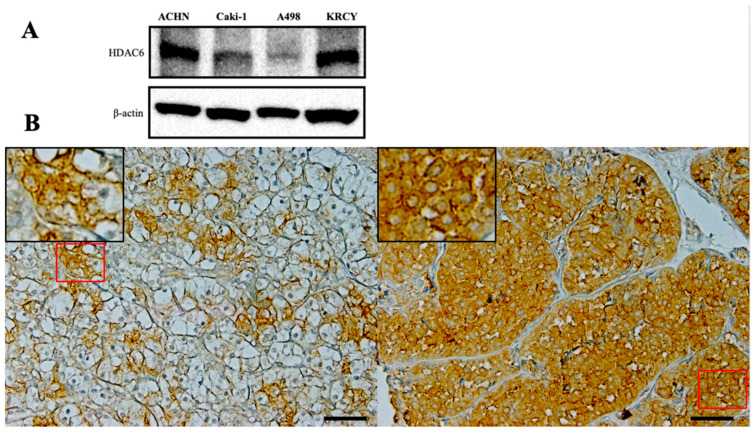
HDAC6 expression of renal cancer cell lines and renal cancer tissues. (**A**) Western blot analysis demonstrating the expression of HDAC6 in RCC cell lines. β-Actin is used as the loading control. (**B**) Immunohistochemical staining of clear-cell (**left**) and non-clear-cell (**right**) RCC against HDAC6. HDAC6 is localized predominantly in the cytoplasm. The regions highlighted by red squares are magnified for a detailed view. The scale bar indicates 50 μm. HDAC6, histone deacetylase 6; RCC, renal cell carcinoma.

HDAC6 expression in the RCC cell lines was confirmed using immunoblotting (Figure 1A). In the tissue microarray, 3 of the 27 cases could not be evaluated due to the small amount of cancer tissue. We evaluated the remaining 24 cases, of which the number of Fuhrman grade 1, 2, 3, and 4 patients was 5, 7, 8, and 4, respectively. HDAC6 was detected in the cytoplasm of renal cancer cells, of which approximately 40% showed high expression and approximately 60% showed low expression. High HDAC6 expression was found in 60% (3 of 5) of grade 1 tumors, 14.3% (1 of 7) of grade 2 tumors, 62.5% (5 of 8) of grade 3 tumors, and 25% (1 of 4) of grade 4 tumors (Table 1), with no statistical difference between Fuhrman grades. Representative images of clear-cell and non-clear-cell RCC specimens with high HDAC6 expression are shown in Figure 1B.

### 3.2. HDAC6 Inhibition Suppressed the Survival and DNA Replication of RCC Cells

**Figure 2:**
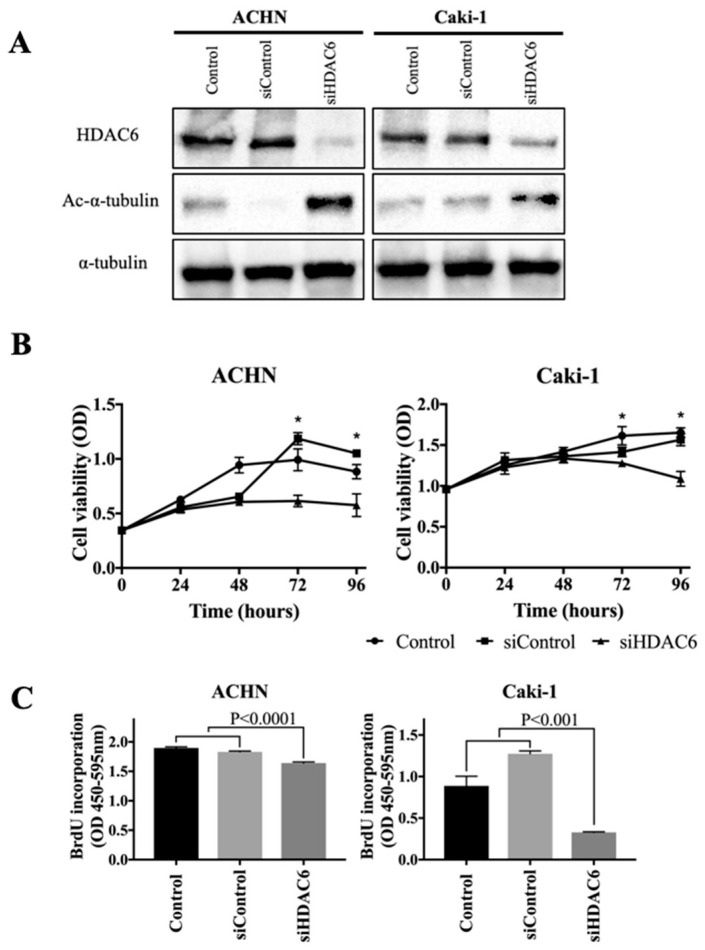
Results of the HDAC6 knockdown using siRNA. (**A**) Western blot analysis illustrating the decrease in HDAC6 expression and the increase in acetyl-α-tubulin expression following transfection with siRNA targeting HDAC6 (siHDAC6) in ACHN and Caki-1 cells compared to untransfected controls or cells transfected with negative control siRNA (siControl) for 48 h. (**B**) Knockdown of HDAC6 decreases the viability of RCC cells. Cell viability is measured by the MTS assay at 24, 48, 72, and 96 h. The siHDAC6 group was compared to the control or siControl group. An asterisk marks (*) indicate statistically significant differences (*p* < 0.05). (**C**) Knockdown of HDAC6 decreases the DNA replication of RCC cells. DNA replication is measured by the BrdU incorporation assay at 72 h. Differences are analyzed by a one-way ANOVA. HDAC6, histone deacetylase 6; RCC, renal cell carcinoma; BrdU, 5-bromo-2-deoxyuridine; OD, optical density.

First, we performed knockdown of HDAC6 using siRNA to investigate the effect of HDAC6 inhibition on RCC cells. Knockdown of HDAC6 was confirmed by immunoblotting, as indicated by decreased expression of HDAC6 and increased expression of acetyl-α-tubulin (Figure 2A). As shown in Figure 2B,C, knockdown of HDAC6 decreased the viability and DNA replication of RCC cells.

**Figure 3:**
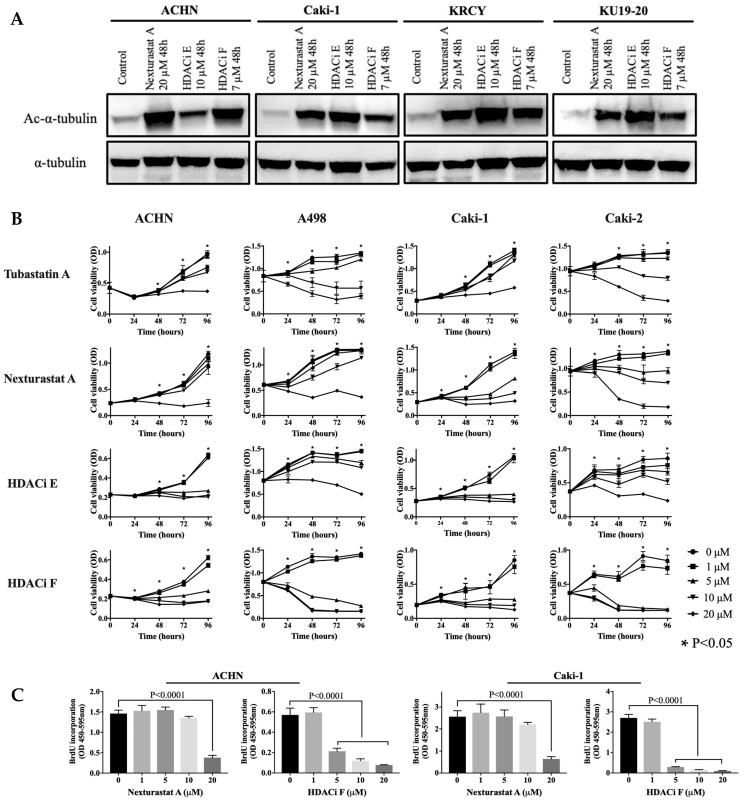
Results of the HDAC6 inhibition using small-molecule selective HDAC6 inhibitors. (**A**) Western blot analysis of RCC cells, untreated control or treated with HDAC6 inhibitors for 48 h. HDAC6 inhibitors suppress HDAC6 activity in RCC cells, as indicated by the increased expression of acetyl-α-tubulin. (**B**) Selective HDAC6 inhibitors decrease the viability of RCC cells in a concentration-dependent manner. Cell viability is measured by the MTS assay in these cells at 24, 48, 72, and 96 h. The results of four representative inhibitors are shown. An asterisk marks (*) indicate statistically significant differences (*p* < 0.05). (**C**) Selective HDAC6 inhibitors decrease DNA replication in RCC cells. DNA replication is measured by the BrdU incorporation assay in these cells at 48 h. HDAC6, histone deacetylase 6; RCC, renal cell carcinoma; BrdU, 5-bromo-2-deoxyuridine; OD, optical density.

Next, we confirmed that three representative HDAC6 inhibitors with lower IC50s suppressed HDAC6 activity, as indicated by increased expression of acetyl-α-tubulin (Figure 3A). Using the MTS assay, we evaluated the ability of 12 selective HDAC6 inhibitors to suppress survival in RCC cells. As shown in Figure 3B and Figure S1 and Table 2 [19,20,21,22,23,24,25,26,27], treatment with these inhibitors decreased the viability of RCC cells in a concentration-dependent manner, with IC50 values in the low micromolar concentration range for most drugs. The results of the BrdU incorporation assay demonstrated that treatment with nexturastat A and HDACi F inhibited the DNA replication of RCC cells compared with the respective control groups (Figure 3C).

### 3.3. HDAC6 Inhibition-Induced Apoptosis in RCC Cells

**Figure 4:**
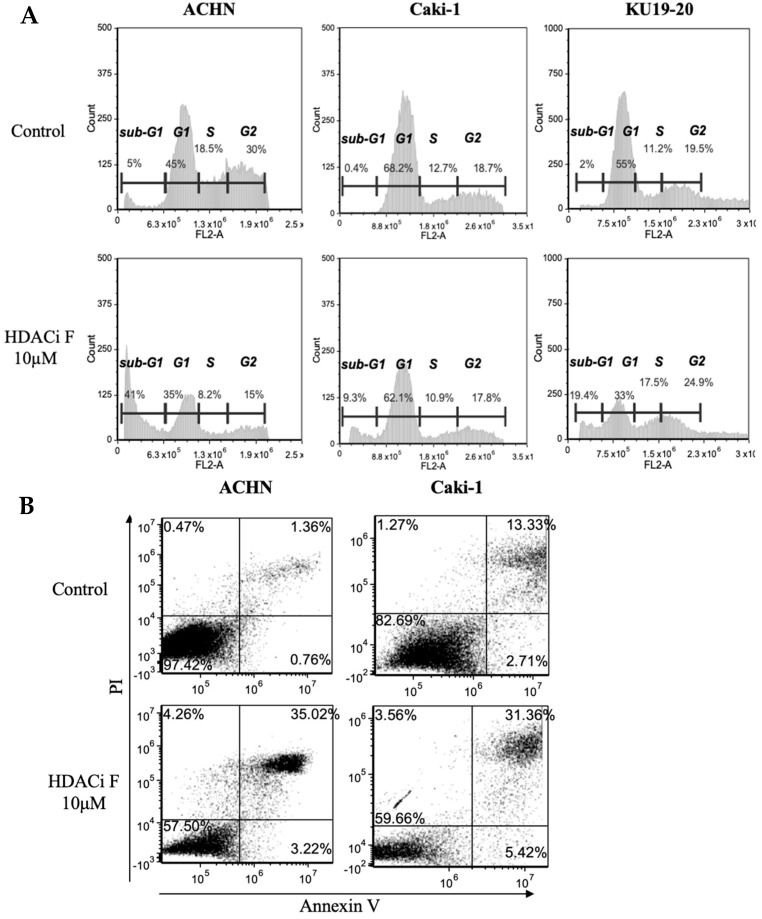
Effects of HDAC6 inhibition on cell cycle alterations and apoptosis. (**A**) Results of PI flow cytometry analyses of RCC cells treated with 10 μM of HDACi F for 48 h are shown. Selective histone deacetylase 6 (HDAC6) inhibitors induce apoptosis (increased sub-G1 cell population) in RCC cells. FL2-A represents the total cell fluorescence area. (**B**) Results of Annexin V–PI flow cytometry analyses of RCC cells treated with 10 μM of HDACi F for 48 h are shown. The viable cells and late apoptotic/secondary necrotic cells are represented by the lower left quadrant (Annexin V negative/PI negative) and upper right quadrant (Annexin V positive/PI positive), respectively. PI, propidium (PI) iodide; RCC, renal cell carcinoma.

PI staining flow cytometry revealed that treatment with HDACi F for 48 h induced apoptosis, as indicated by the increased sub-G1 cell population in ACHN, Caki-1, and KU19-20 cells compared with the respective control groups (Figure 4A). Furthermore, treatment with HDACi F for 48 h increased the Annexin V/PI double-positive cell population, indicating late-stage apoptosis (Figure 4B).

## 4. Discussion

The acetylation status of histones, a fundamental aspect of epigenetic regulation, intricately controls the dynamics of gene expression. Within this regulatory framework, histone acetylation assumes a pivotal role, finely regulated by two opposing enzymes: HDACs and histone acetyltransferases (HATs). HDACs are classified into four classes based on their structure and function. Class I HDACs, primarily localized in the nucleus, include HDAC1, 2, 3, and 8. Class II HDACs are further divided into two subclasses: IIa (HDAC4, 5, 7, and 9) and IIb (HDAC6 and 10), each shuttling between the nucleus and cytoplasm. Class III HDACs, also known as sirtuins (SIRT1-7), requiring nicotinamide adenine dinucleotide (NAD+) for their deacetylase activity, are primarily localized in the nucleus, cytoplasm, and mitochondria. Finally, class IV consists of a single member, HDAC11, predominantly found in the nucleus, sharing sequence similarity with both class I and II HDACs [28]. HDACs, primarily responsible for removing acetyl groups from histone tails, contribute to chromatin compaction, thereby repressing gene transcription. Conversely, HATs catalyze the addition of acetyl groups, leading to chromatin relaxation and activation of gene expression [8]. This delicate balance between HDAC and HAT activities is essential for maintaining proper cellular function and homeostasis. However, dysregulation in this equilibrium can have profound implications, creating an environment conducive to carcinogenesis and cancer progression [29]. Understanding the distinct localization and structural characteristics of HDAC classes is essential for elucidating their diverse roles in cellular physiology and pathology, thereby facilitating targeted therapeutic interventions in various diseases, including cancer. Recognizing the pivotal role of HDAC dysregulation in driving oncogenic processes, HDACs have emerged as promising targets for anticancer therapeutics. Inhibiting HDAC activity offers the potential to restore the balance of histone acetylation, thereby reprogramming aberrant gene expression profiles and impeding tumor growth. The pursuit of HDAC-targeted therapies represents a cutting-edge frontier in cancer treatment, providing an opportunity to leverage epigenetic modifications for therapeutic benefit and improve patient outcomes.

To date, five HDAC inhibitors have been approved by the United States Food and Drug Administration: vorinostat (SAHA), belinostat, pabinostat, romidepsin, and tucidinostat. Among these, the former three are categorized as pan-HDAC inhibitors, while romidepsin and tucidinostat target class I and class I/II HDACs, respectively [30]. These HDAC inhibitors were initially shown to be effective for hematological malignancies rather than solid tumors [7]. Recent reports have highlighted the efficacy of tucidinostat against breast cancer [31]. However, a more significant concern lies in the serious adverse effects associated with these HDAC inhibitors, including hematotoxicity, gastrointestinal toxicity, and cardiotoxicity. Isoform-selective HDAC inhibitors, which do not broadly inhibit many HDAC isoforms, might reduce the toxicities associated with pan-HDAC inhibitors. HDAC6 belongs to class II HDACs, which can move between the cytoplasm and the nucleus. Unlike other HDACs, HDAC6 primarily localizes to the cytoplasm and regulates the acetylation of various cytoplasmic proteins rather than engaging in epigenetic gene control via histone acetylation [32]. HDAC6 knockout mice are viable without major physiological dysfunction, whereas HDAC1-, HDAC2-, and HDAC3-knockout mice all die prenatally or immediately after birth [9,33]. These facts suggest that selective HDAC6 inhibitors may have mild side effects, consistent with the favorable safety profile observed for the selective HDAC6 inhibitor ricolinostat in patients with relapsed/refractory lymphoma [34].

We confirmed that HDAC6 was expressed in renal cancer cell lines using Western blot and immunohistochemistry. HDAC6 plays a crucial role in cancer initiation, progression, proliferation, metastasis, and maintenance of cancer stem cells through the acetylation of cytoplasmic proteins [10,11], thereby contributing to cancer promotion. HDAC6 regulates multiple processes controlled by heat shock protein 90 (Hsp90), a non-histone substrate and molecular chaperone critical for protein folding and the degradation of misfolded proteins. HDAC6 controls tumor cell proliferation and apoptosis through its interaction with Hsp90. Additionally, HDAC6 prevents apoptosis in cancer cells by regulating Chk1, a cell cycle checkpoint protein, and Ku70, a non-histone protein involved in DNA damage repair [35]. These mechanisms partially explain how HDAC6 inhibition leads to decreased cell viability and the induction of apoptosis in renal cancer cells. The correlation between HDAC6 expression and the prognosis of RCC patients was investigated in a previous study involving 45 fresh RCC samples and 132 paraffin-embedded tissues, along with their corresponding adjacent non-tumor tissues [36]. The study found a significant upregulation of HDAC6 mRNA expression in RCC tissues compared to adjacent non-tumor tissues. Moreover, the study revealed a correlation between HDAC6 expression levels and histologic grade as well as overall survival among RCC patients. Patients with high HDAC6 expression exhibited poor survival outcomes, with high HDAC6 expression emerging as an independent poor prognostic factor for RCC patients. These findings are consistent with the cancer-promoting effects of HDAC6, providing preliminary evidence supporting the exploration of HDAC6 inhibitors as a novel therapeutic strategy for RCC.

Notably, combination therapies involving HDAC6 inhibitors have garnered significant attention in recent years. HDAC6 inhibitors not only exert direct anti-tumor effects but also hold promise for synergistic effects and overcoming drug resistance when used in combination with other therapeutic modalities. For instance, HDAC6 inhibitors have been demonstrated to enhance the sensitivity of cancer cells to various chemotherapeutic agents such as temozolomide [37], gemcitabine [38,39], oxaliplatin [38], eribulin [40], WEE1 inhibitor adavosertib [41], 5-FU [42], and imatinib [43]. It has also been reported that HDAC6 inhibition enhances the radiosensitivity of cancer cells in lung cancer, glioma, and bladder cancer [11,44,45,46]. HDAC6 is closely associated with resistance to sorafenib and gefitinib in lung cancer cells [47,48], resistance to microtubule-targeting anticancer drugs in melanoma cells [49], tamoxifen resistance in breast cancer cells [50], and bortezomib resistance in multiple myeloma cells [51]. One contributing factor to HDAC6-mediated drug resistance is its association with autophagy [52]. HDAC6 plays a pivotal role in orchestrating autophagy by facilitating the transport of ubiquitinated protein aggregates for clearance. Furthermore, autophagy enhances the survival of cancer cells under stress conditions, thereby promoting chemotherapeutic resistance in cancer. Inhibition of HDAC6 prevents the fusion of autophagosomes with lysosomes, leading to the accumulation of protein aggregates and subsequent apoptotic cell death. Thus, HDAC6 inhibition holds the potential to overcome drug resistance via autophagy inhibition, making it a promising target for combination therapy strategies.

HDAC6 is also known to modulate the tumor immune microenvironment. In lung cancer, it has been reported that the high HDAC6 expression group exhibits a significantly poorer response to immunotherapy and significantly shorter progression-free survival compared to the low expression group [53]. HDAC6 is involved in PD-L1 expression through the STAT3 pathway, and its inhibition results in a reduction in PD-L1 expression in various types of cancer [53,54,55,56,57,58]. Furthermore, HDAC6 inhibition enhanced the sensitivity of cancer cells to anti-PD-L1 blockade by reducing the number of M2 macrophages, which negatively regulate the immune response [59]. Additionally, HDAC6 inhibition also decreases the immunosuppressive cytokine IL-10 and activates the responsiveness of antigen-specific naïve T cells and CD4+ T cells [60]. Therefore, the combination of HDAC6 inhibition and immunotherapy may hold promise as an effective approach. Preclinical studies have demonstrated that HDAC6 inhibition enhances the efficacy of anti-PD-1/PD-L1 immunotherapy in cancers such as melanoma [61,62], ovarian cancer [63], chronic lymphocytic leukemia [57], and colorectal cancer [56]. Many preclinical and clinical studies have investigated the efficacy of various combination therapies, including HDAC6 inhibitors alongside proteasome inhibitors, immune checkpoint inhibitors, and chemotherapeutic agents. Such combination strategies aim to leverage the complementary mechanisms of action of the drugs involved, thereby enhancing therapeutic efficacy while minimizing adverse effects. As a prime example, the combination of the selective HDAC6 inhibitor ricolinostat with bortezomib demonstrated promising efficacy and safety in phase I/II trials for multiple myeloma [64].

Cell motility is pivotal in cancer metastasis, which is the most lethal event in tumorigenesis. HDAC6 regulates the stability of microtubules through tubulin acetylation, thereby controlling cell motility [32,60]. In addition to its role in microtubule-dependent cell motility, HDAC6 also acts on another non-histone substrate, cortactin. Acetylation of cortactin by HDAC6 inhibition reduces actin-dependent cell motility. Furthermore, Hsp90 is essential for the stability and function of proteins involved in tumor metastasis [60]. The inhibition of HDAC6 leads to the acetylation of Hsp90, resulting in a decrease in its substrate proteins. These provide the theoretical basis for the efficacy of HDAC6 inhibitors in preventing cancer metastasis. Previous studies revealed that HDAC6 inhibitors showed anti-metastatic effects in pancreatic and breast cancer cells in vitro and in vivo [65,66]. This therapeutic benefit may be further enhanced by combination with other therapeutic agents.

In the present study, we utilized a panel of 12 HDAC6 inhibitors and demonstrated that selective HDAC6 inhibition decreased RCC cell survival by inducing apoptosis. Generally, HDAC inhibitors consist of the following three main motifs: a cap group that interacts with the enzyme’s surface, a zinc-binding group that binds to the catalytic domain, and a linker group that connects these two parts [21]. An appropriately optimized cap group can improve both efficacy and selectivity by effectively interacting with the residues on the enzyme’s surface [21]. The zinc-binding group functions by binding to the active site of HDAC and inhibiting its enzymatic activity; in most of the 12 HDAC6 inhibitors we used, the hydroxamate group (-CO-NHOH) is utilized. Tubastatin A and Nexturastat A are selective HDAC6 inhibitors reported by Kozikowski et al. in 2010 and 2012 [19,20], respectively. These are the most extensively studied HDAC6 inhibitors in the field of cancer research, following ricolinostat. HDACi A, developed based on Nexturastat A, has been shown to suppress melanoma tumor growth by inducing an immune response through the downregulation of IL-10 [21]. HDACi B, C, and D were developed with structural scaffolding from Tubastatin A, utilizing a bicyclic cap to achieve superior selectivity over HDAC1 [22]. These inhibitors have demonstrated efficacy in in vitro models for Charcot-Marie-Tooth disease, a hereditary peripheral neuropathy affecting both motor and sensory functions. HDACi E and F share highly similar structures, with structural similarities to Tubastatin A, and exhibit dual effects of inhibiting HDAC6 as well as activating both Nrf2 and HIF-1α [23]. Activation of Nrf2 and HIF-1α exerts neuroprotective effects in models of neurodegenerative diseases such as Parkinson’s disease, Alzheimer’s disease, and ischemic stroke. HDACi G is a selective HDAC6 inhibitor containing isoxazole-3-hydroxamate as the zinc-binding group and linker group. It has been suggested to exhibit immune-related antitumor activity by increasing the infiltration of CD8+ and NK+ T cells and the ratio of M1 to M2 macrophages in the tumor microenvironment [25]. HDACi H, with an aminoquinoline cap group, is a non-hydroxamate HDAC6 inhibitor that demonstrated over 3000-fold selectivity for HDAC6 over HDAC1 [26]. HDACi H has been shown to exert anti-inflammatory effects by modulating the function of Foxp3+ regulatory T cells in a mouse model. HDACi I and J are non-hydroxamate HDAC6 inhibitors designed to avoid the genotoxicity commonly associated with hydroxamates and are optimized for central nervous system disorders with enhanced brain accessibility [27]. Although these inhibitors have not been used previously in other cancers or clinical trials, they have been confirmed to inhibit HDAC6 activity selectively, and their structures are shown in Table 2. It is imperative to further optimize and investigate these inhibitors for their clinical applications.

The major limitation of our study is the lack of in vivo experiments. Furthermore, it would have been beneficial to include analyses of cell invasion and migration, as well as experiments investigating the effects of HDAC6 inhibition using three-dimensional cell cultures. Although it is essential to validate these findings through in vivo studies to elucidate the translational potential of HDAC6 inhibition in clinical settings, our experimental results demonstrate the therapeutic promise of HDAC6 inhibitors as potent anticancer agents against RCC. A deeper understanding of the molecular mechanisms underlying the anticancer effects of HDAC6 inhibition will help improve their therapeutic application and optimize treatment strategies for RCC. Ultimately, by utilizing the therapeutic power of HDAC6 inhibition, we may pave the way for novel and more effective therapeutic interventions to combat metastatic RCC and improve patient outcomes.

## 5. Conclusions

The results of this study demonstrate the anticancer effects of selective HDAC6 inhibitors on RCC and support future clinical trials of such inhibitors for advanced RCC patients.

## Figures and Tables

**Table 1:** Summary of the tissue microarray results for HDAC6 expression.

Fuhrman grade	1	2	3	4
High HDAC6 expression	3	1	5	1
Low HDAC6 expression	2	6	3	3

**Table 2:** The structures and the IC50s of HDAC6 inhibitors.

Compound	Structure	ACHN	A498	Caki-1	Caki-2	KRCY
Tubastatin A [19]	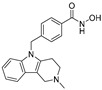	26.9 (20–36.2)	9.8 (7.7–12.4)	16.8 (13.4–21)	14.3 (10.5–19.4)	21.4 (17.1–26.9)
Nexturastat A [20]	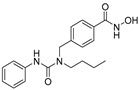	18.5 (13.5–25.6)	21.6 (20–27.6)	5.4 (4.2–6.7)	9.1 (7–11.8)	17.4 (11.9–25.4)
HDACi A [21]	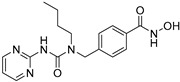	Not converged	484 (195–1202)	108 (78.7–148)	365 (223–598)	1021 (291–3574)
HDACi B [22]	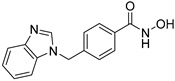	27.8 (23.7–32.5)	32.1 (20.8–49.6)	31.5 (23.4–42.5)	28.1 (19.3–41)	29 (20.3–41.6)
HDACi C [22]	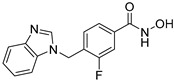	29.2 (23.9–35.8)	27.2 (19.3–38.3)	30.3 (19.8–46.3)	27.5 (19.7–38.3)	29.9 (20.4–43.7)
HDACi D [22]	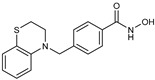	22.6 (17.3–29.4)	12.5 (10.9–14.4)	12.4 (9–17.1)	20 (15.5.–25.8)	20.5 (14.6–28.8)
HDACi E [23,24]	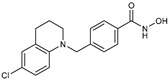	21.7 (17–27.8)	24.3 (17.3–34.1)	8.2 (6.8–10)	16.9 (13.9–20.5)	6.1 (4.8–7.6)
HDACi F	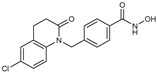	11.1 (8.4–14.7)	3.1 (2.2–4.3)	3.4 (2.6–4.3)	2.2 (1.6–3.1)	5.6 (3.7–8.7)
HDACi G [25]	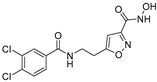	25.4 (15.7–40.1)	61 (39.5–94.3)	9.2 (7–12.1)	142 (94.1–216)	60.1 (36.7–98.5)
HDACi H [26]	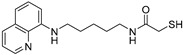	47.6 (29.4–77.2)	44.4 (25–79)	50.2 (26.6–94.8)	42 (25.1–70.2)	52.9 (29–96.3)
HDACi I [27]	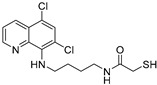	36.6 (26.8–49.8)	83.6 (63.2–110)	46.3 (33.8–63.5)	54 (37.6–77.6)	27 (16.8–43.4)
HDACi J [27]	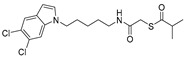	145.9 (99–215)	76.5 (58–101)	56.9 (41.4–78.2)	39.5 (26.6–58.7)	60.9 (38.4–96.5)

The 95% confidence intervals are shown in parentheses. IC50 is the drug concentration that inhibited the viability of cancer cells by 50%.

## Data Availability

The data used in this study are available from the corresponding author upon reasonable request.

## Supplementary Materials

The following supporting information can be downloaded at: https://www.mdpi.com/article/10.3390/jpm14070704/s1, Figure S1: Results of MTS assay for HDAC6 inhibition in RCC cell lines using a panel of 12 selective HDAC6 inhibitors.
