# Supplementary figures and images for: Selective HDAC6 Inhibition Has the Potential for Anti-Cancer Effect in Renal Cell Carcinoma
Source: J Pers Med. 2024 Jun 30;14(7):704. doi: 10.3390/jpm14070704 (PMC11278056; doi:10.3390/jpm14070704)

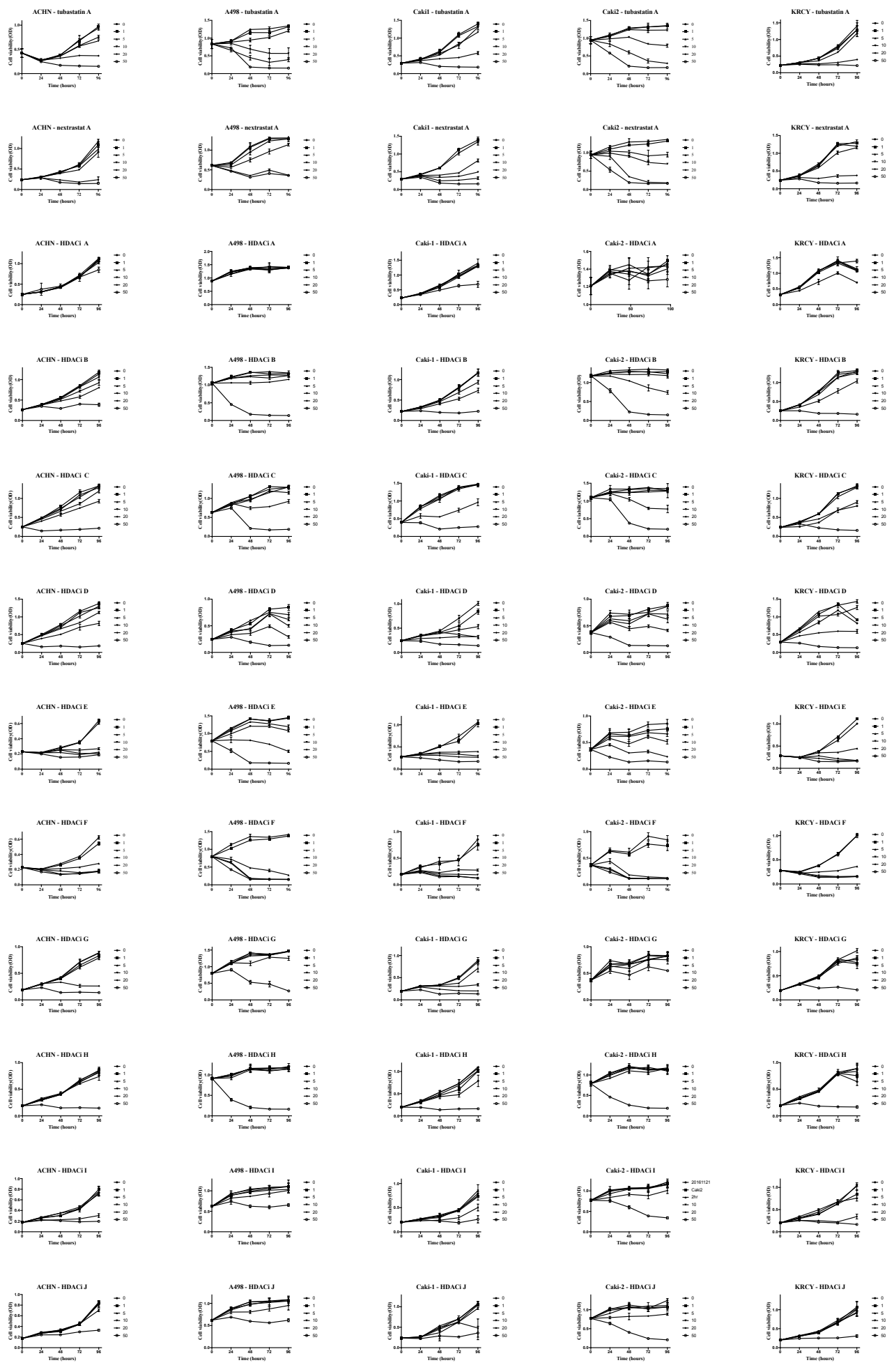

Supplement: Supplementary file 1 [file jpm-14-00704-s001.zip › jpm-3031525-supplementary.pdf]
